# Efficiency Enhancement with the Ferroelectric Coupling Effect Using P(VDF‐TrFE) in CH_3_NH_3_PbI_3_ Solar Cells

**DOI:** 10.1002/advs.201900252

**Published:** 2019-07-04

**Authors:** Endong Jia, Dong Wei, Peng Cui, Jun Ji, Hao Huang, Haoran Jiang, Shangyi Dou, Meicheng Li, Chunlan Zhou, Wenjing Wang

**Affiliations:** ^1^ The Key Laboratory of Solar Thermal Energy and Photovoltaic System Institute of Electrical Engineering Chinese Academy of Sciences Beijing 100190 P. R. China; ^2^ University of Chinese Academy of Sciences (UCAS) Beijing 100049 P. R. China; ^3^ State Key Laboratory of Alternate Electrical Power System with Renewable Energy Sources School of Renewable Energy North China Electric Power University Beijing 102206 P. R. China

**Keywords:** ferroelectric coupling effect, perovskite solar cells, semi‐transparent

## Abstract

A novel ferroelectric coupling photovoltaic effect is reported to enhance the open‐circuit voltage (*V*_OC_) and the efficiency of CH_3_NH_3_PbI_3_ perovskite solar cells. A theoretical analysis demonstrates that this ferroelectric coupling effect can effectively promote charge extraction as well as suppress combination loss for an increased minority carrier lifetime. In this study, a ferroelectric polymer P(VDF‐TrFE) is introduced to the absorber layer in solar cells with a proper cocrystalline process. Piezoresponse force microscopy (PFM) is used to confirm that the P(VDF‐TrFE):CH_3_NH_3_PbI_3_ mixed thin films possess ferroelectricity, while the pure CH_3_NH_3_PbI_3_ films have no obvious PFM response. Additionally, with the applied external bias voltages on the ferroelectric films, the devices begin to show tunable photovoltaic performance, as expected for the polarization in the poling process. Furthermore, it is shown that through the ferroelectric coupled effect, the efficiency of the CH_3_NH_3_PbI_3_‐based perovskite photovoltaic devices is enhanced by about 30%, from 13.4% to 17.3%. And the open‐circuit voltages (*V*_OC_) reach 1.17 from 1.08 V, which is reported to be among the highest *V*_OC_s for CH_3_NH_3_PbI_3_‐based devices. It should be noted in particular that the thickness of the layer is less than 160 nm, which can be regarded as semi‐transparent.

The power conversion efficiency (PCE) of hybrid organic–inorganic halide perovskite solar cells (PSCs) has entered the over 22% era. Many conventional approaches to enhancing the PCE of the PSCs layer have been reported. These approaches include controlling the crystalline process of the perovskite layer (e.g., increasing the grain size to extend the lifetime of the minority carriers),^1^, ^2^, ^3^, ^4^, ^5^, ^6^ aligning the energy levels (e.g., reducing the work function for the downward vacuum level at the interfaces of ETL/perovskite layer and perovskite/HTL)^7^, ^8^, ^9^, ^10^, ^11^ and providing chemical stability for the moisture,^12^, ^13^, ^14^ and so on. Aside from those strategies, the ferroelectric coupling photovoltaic effect, in which an additional internal electric field induced by the unscreened polarization charges of introduced ferroelectric materials can increase the photogenerated‐carrier collection efficiency,^15^, ^16^ is a distinctive routine for the enhancement of the efficiency. This routine has also been attempted for organic photovoltaic (OPV) devices before.^17^, ^18^, ^19^, ^20^, ^21^, ^22^

Although the excitons in CH_3_NH_3_PbI_3_ perovskite layers have extremely low binding energy and are easy to separate at room temperature,^23^, ^24^, ^25^ the charge extraction efficiency can also be promoted by the built‐in field adhibiting from the ferroelectric polarization.^21^, ^22^ The ferroelectric polymer poly(vinylidenefluoride‐trfluoroethylene) (P(VDF‐TrFE)) has proven to be effective in increasing the charges' transfer excitons (CTEs) separation and the charge extraction and collection efficiency in ferroelectric organic photovoltaic (FE‐OPV) devices.^18^, ^19^, ^20^, ^21^, ^22^ Additionally, PVDF was also confirmed to be an effective additive for increasing the quality of CH_3_NH_3_PbI_3_ films from the perspective of increasing the crystallinity and the passivation of the grain boundary.^26^, ^27^ Therefore, P(VDF‐TrFE) could be a proper ferroelectric polymer for achieving the goal of enhancing PCEs in PSCs via the ferroelectric coupling photovoltaic effect. In this research, we incorporated the ferroelectric coupling photovoltaic effect using a permanent electric field with a ferroelectric copolymer dopant inside absorber layer to increase the charge separation and the extraction between the electrode and the semiconductor layer, so that it could enhance the solar power conversion efficiency.

In this study, the ferroelectric photovoltaic coupling effects on the performance of hybrid P(VDF‐TrFE)/CH_3_NH_3_PbI_3_ planar heterojunction solar cells were investigated. The access of ferroelectric P(VDF‐TrFE 50/50) doping into the CH_3_NH_3_PbI_3_ layer was achieved by a simple solvent extraction method. The ferroelectricity of the crystalline β‐phase P(VDF‐TrFE) obtained by this method was measured experimentally. To the best of our knowledge, this is the first time that ferroelectric polymers have been added to the active absorption layer of PSCs while retaining their ferroelectricity and proving to be applicable to PSCs. In addition, this semiconducting ferroelectric blend layer (FBL) exhibited outstanding photovoltaic properties. The average open‐circuit voltage (*V*_oc_) of the FE‐PSCs with FBL achieved a value of over 1.17 V. The highest *V*_oc_ value reached 1.174 V, which is among the highest values reported for CH_3_NH_3_PbI_3_‐based solar cells.

The molecular model structure of the P(VDF‐TrFE) copolymer applied in this study is shown in **Figure** **1**a. The molecular chains were perpendicular to the direction of the dipole and all dipoles along the chain, which was a result of the electron affinity difference of the CF_2_ and CH_2_ groups. White P(VDF‐TrFE) polymer powder and precursor PbI_2_ and MAI powder are illustrated in Figure S1a in the Supporting Information. P(VDF‐TrFE) and PbI_2_ both had good solubility in dimethyl formamide (DMF), while even slight amount of P(VDF‐TrFE) (less than 0.1% weight ratio to DMF) could not be dissolved in higher PbI_2_ concentration DMF solvent (more than 0.7 m), as shown in Figure S1b in the Supporting Information. This was due to the strong interaction between the nucleophile solvent DMF and PbI_2_. With this consideration, the concentration of perovskite precursor was adjusted below 0.65 m. Meanwhile, antisolvent diethyl aether, as an effective extract solvent for DMF, both for perovskite and P(VDF‐TrFE), was used in the FBL synthesis process. The details of the doping method are described in Experimental Section in the Supporting Information.

**Figure 1:**
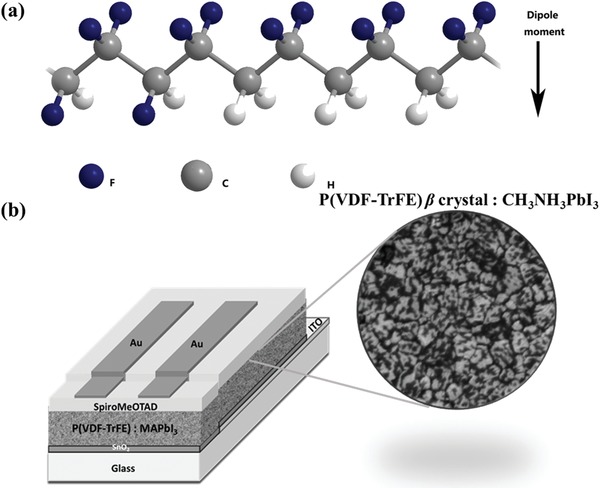
a) Chemical Structure of P(VDF‐TrFE). b) Structure of the P(VDF‐TrFE) incorporated into the PSC device and the FBL morphology.

The as‐grown P(VDF‐TrFE) phase was supposed to be annealed above its Curie temperature of 65 °C,^28^, ^29^ to ensure its crystalline ferroelectric property. The annealing process was also compatible and necessary for the CH_3_NH_3_PbI_3_ crystalline film. **Figure** **2**f shows the (110)‐(200)^30^, ^31^ peaks demonstrated using the X‐ray diffraction (XRD) measurements. The lattice spacing for the d(110) peak was calculated as 0.467 nm from the peak location with Bragg's Law, as shown by Equation (1) shows, which agreed with previous reported^32^ values of β‐phase P (VDF‐TrFE)

(1) 2d sinθ= λ

**Figure 2:**
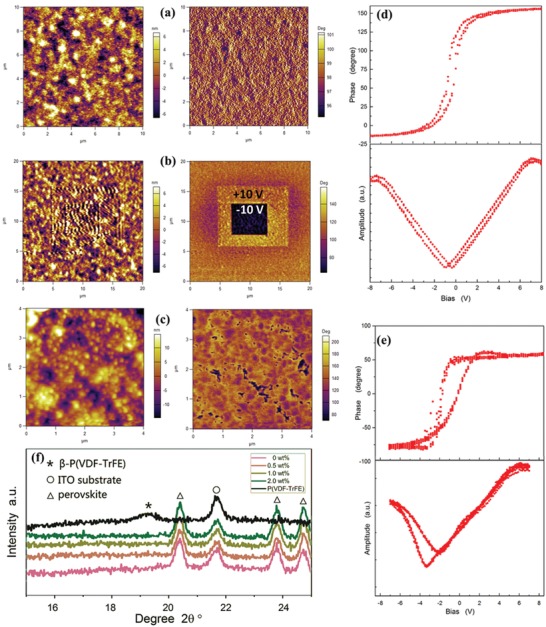
a) AFM topology and PFM in‐phase images of the origin P(VDF‐TrFE) film. b) AFM topology and PFM in‐phase images of the localizing polarized P(VDF‐TrFE) film. c) AFM topology and PFM in‐phase images of P(VDF‐TrFE) doped CH_3_NH_3_PbI_3_ film. d) Nonlinear hysteresis loop (phase and amplitude) of pure P(VDF‐TrFE) film. e) Nonlinear hysteresis loop (phase and amplitude) of P(VDF‐TrFE) doped CH_3_NH_3_PbI_3_ film. f) XRD of the P(VDF‐TrFE) and CH_3_NH_3_PbI_3_ doped with various concentrations of P(VDF‐TrFE) films.

The relatively weak d(110) peak (marked as *) implied the poor crystallinity of the polymer P(VDF‐TrFE), which may have been caused by the strong interactions between the DMF molecule and the CF_2_ groups in the crystalline process. Therefore, the ferroelectricity of the P(VDF‐TrFE) that crystallized in the DMF had to be verified. The polarization hysteresis loop (*P–E*) loop method was addressed with piezoresponse force microscopy (PFM) to measure the pure P(VDF‐TrFE) film fabricated with the DMF solvent and the same crystalline process. Figure 2b shows a local response of the phase and amplitude to a bias electric field on the pure P(VDF‐TrFE) film surface, which directly revealed the hysteretic property of such films. An intuitive image of the switchable polarization behaviors was revealed as shown in Figure 2c. A bias voltage of *V*_tip_ = 10 V was applied to a 10 × 10 µm^2^ square area in the center of the scan field, then a bias voltage of *V*_tip_ = −10 V was applied to an area of 5 × 5 µm^2^ near the center. The reversal phase evidence verified the ferroelectricity of the P(VDF‐TrFE) thin film as well. The FBL was also investigated via the same method as that presented in Figure 2e. The FBL showed a similar PFM response on the P(VDF‐TrFE) gathering places in Figure 2c. As mentioned in the Supporting Information, the dopant P(VDF‐TrFE) was easily gathered under a higher concentration over 2 wt%. Therefore, we controlled the concentration of the doping concisely. In Figure 2c, the roughness was ≈10 nm on the FBL surface (2 wt% sample), and it was completely different from the roughness on the pure P(VDF‐TrFE) film surface (5 nm).

In contrast, we found no clear hysteresis for the *P–E* loops on the pure CH_3_NH_3_PbI_3_ films, nor on the areas where there was no P(VDF‐TrFE) gathered on the FBL (see Figure S2 in the Supporting Information). Therefore, in this study we considered the CH_3_NH_3_PbI_3_ materials to have no obvious ferroelectricity, which also agreed with other previous reported research..^33^, ^34^ However, it must be admitted there are different academic opinions about whether CH_3_NH_3_PbI_3_ has ferroelectricity.^35^, ^36^, ^37^ Therefore, in this study, the ferroelectricity of the FBL that was fabricated with the Supporting Information method was only caused by the P(VDF‐TrFE) dopants inside.

To predict the possible potential contribution to the performance for the polarization internal field induced by the ferroelectric material P(VDF‐TrFE) in FBL, the interaction mechanism of the doping ferroelectric material in FE‐PSCs needed to be studied using semiconductor physics theory. Based on the classic dipole‐electric field model,^19^ the local additional internal electrical field (*E*_add_), caused by the ferroelectric P(VDF‐TrFE) dispersed in perovskite photoactive layer, can be described as

(2) $$E_{\mathrm{add}}=\frac{4\pi \sigma_{P}}{\varepsilon_{0}\varepsilon_{\mathrm{fe}}}\;f$$

where σ_P_ is the pyroinduced surface charge density, *f* is the volume fraction occupied by the dipoles, and ε_fe_ is the relative dieletric constant of P(VDF‐TrFE). According to the density and mass ratio of CH_3_NH_3_PbI_3_ to P(VDF‐TrFE), *f* was estimated to be ≈10^−3^. By using the nominal remnant polarization of 6 µCcm^−2^ for P(VDF‐TrFE),^38^ the additional internal electric field was calculated to be ≈20 V µm^−1^. In clear contrast, typical PSC devices produce an internal electric field of less than 1.5 V µm^−1^ (calculated from the effective applied potential ≈1 V and an active thickness of ≈0.6 µm), which is much smaller than that of FE‐PSCs. The additional induced electric field can increase both the carrier drift length and the carrier recombination lifetime. For a p–i–n solar cell, *V*_OC_ can be expressed as

(3) $$V_{\mathrm{oc}}=\;nkT/q\times \ln\left(\frac{J_{\mathrm{sc}}}{J_{0}}+1\right)$$

and the Sah–Noyce–Shockley^39^ approximation can be described as

(4) $$J_{0,\mathrm{scr}}=\frac{qn_{i}}{\sqrt{\tau_{e}\tau_{h}}}\;W$$

where *n* is the ideality factor, *k* is the Boltzmann constant, *q* is the elementary charge, *J*_0_ is the reverse saturation current density, *J*_0,scr_ is the reverse saturation space charge region current density, *n*_i_ is the instrinsic carrier concentration, τ_e_ and τ_h_ are the electrons and the hole lifetimes, respectively, and *W* is the width of the depletion region. Since the carrier concentrations of ETL and HTL were much larger than that of perovskite active layer, *W* could be estimated to be about the width of the thickness of the perovskite active layer, i.e., a constant quantity. The reason for this was that the depletion recombination and the bulk/interface/surface recombination were both important in the analysis of photon–electron conversion loss, and the depletion layer was where the ferroelectric dopant layer played a role, as presented in previous reports.^41^, ^42^, ^43^ Therefore, recombinations could occur in the surface, bulk, and depletion regions in such p–i–n structures. Here, the depletion region recombination is what we would like to discuss in detail. Especially for the direct‐bandgap perovskite semiconductor, the depletion or called space charge region current density played a larger role in the dark current density (*J*_dark_) than the diffusion and the radiative current density. Therefore, we could consider *J*_0_ to be *J*_0,scr_ approximately.

(5) $$J_{0}\approx \;J_{0,\mathrm{scr}}=\frac{qn_{i}}{\sqrt{\tau_{e}\tau_{h}}}\;W$$

Thus, the relationship of *V*_OC_ ≈ τ was given approximately by Equations (3) and (5).

The electrons and holes disappeared either via phonon‐assisted nonradiative recombination, with a decay rate constant *k*_f_, or by extraction to charge collecting layers with a field‐dependent dissociation rate, *k*_D_. The probability (*P*) of the physical process by which carriers go through recombination in their lifetime according to the Braun model^40^ can be described as

(6) $$P_{\mathrm{re}}\left(E\right)\;=\frac{k_{f}}{k_{D}\left(E\right)+k_{f}}$$

*k*_D_(*E*) was derived to be

(7) $$k_{D}\;\left(E\right)=k_{R}\;\frac{3}{4\pi a^{3}}e^{-E_{B}/kT}\left[1+b+\frac{b^{2}}{3}+\frac{b^{3}}{18}+\frac{b^{4}}{180}+\cdots \right]$$

and *b* = *e* ^3^ *E*/(8*πε*_0_ε_r_ *k* ^2^ *T* ^2^), where *k*_R_ is the bimolecular rate constant of the bound e–h pair, *a* is the initial separation of the e–h pair at the interface, and *E*_B_ is the binding energy of the e–h pairs. One limiting case of Equation (6) was of interest. As *E* → ∞, *P*_re_(*E*) → 0, which indicated that increasing the built‐in field (*E*) could reduce the probability of recombination (*P*_re_). The reduced *P*_re_ signified that the carrier lifetime (τ) was extended. Then the impact of the built‐in field (*E*) on the *V*_OC_ mechanism was expounded on and the relation diagram was drawn out according to Equations (1), (5), and (6), as shown in **Figure** **3**.

**Figure 3:**
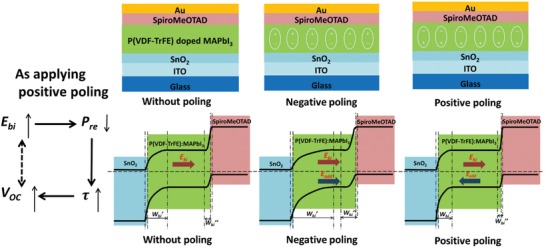
Schematic and band diagram in equilibrium for p–i–n (SnO_2 –_ CH_3_NH_3_PbI_3_ ‐SpiroMeOTAD) solar cells under different poling conditions. The extensions of the respective depletion zones and built‐in voltages are indicated at each side.

After confirming the ferroelectricity of FBL and analyzing the possible contribution of the ferroelectric coupling photovoltaic effect to the performance of the FE‐PSCs, we fabricated a series of FE‐PSC devices with different doping concentrations of P(VDF‐TrFE) in the FBL and tested their performances. **Figure** **4**a shows the top‐view scanning electron microscopy (SEM) images of samples on fully covered glass|ITO|SnO_2_ substrates with different P(VDF‐TrFE) doping concentrations of FBL, which both demonstrates the high crystallization quality of the pure perovskite layer and exhibits the piebald surface morphology of the FBL. Interestingly, the stain‐like pattern was caused by the introduction of P(VDF‐TrFE) and the amount beside the area of darkened spots increased as the P(VDF‐TrFE) doping concentration increased. However, the grain sizes for each situation were considered unaffected by the dopants, which implied that the dopants did not shrink or cause an amorphous phase effect on the perovskite crystalline process.

**Figure 4:**
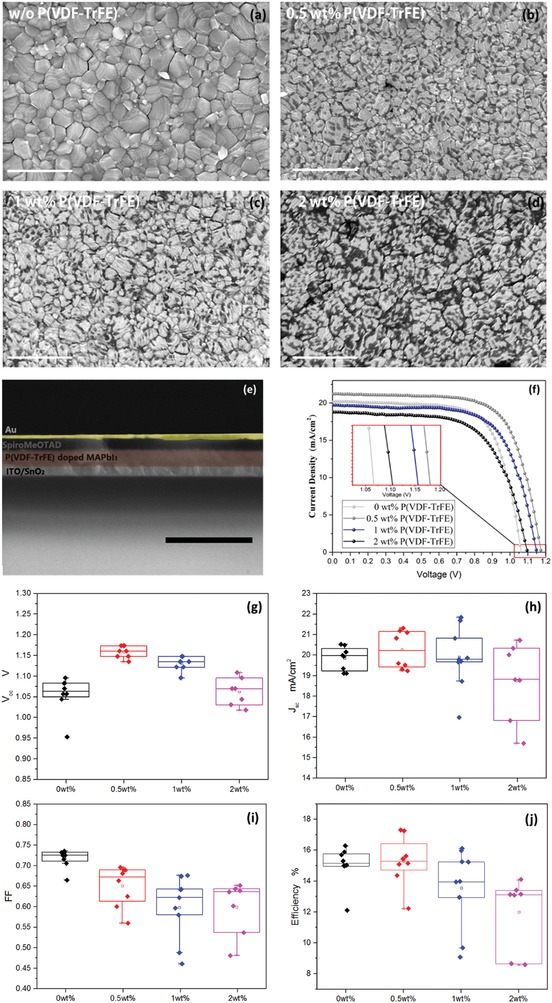
a–d) Top surface morphologies of the CH_3_NH_3_PbI_3_ doped with 0, 0.5, 1, 2 wt% P(VDF‐TrFE) concentrations. e) Cross‐sectional FE‐SEM image of the FE‐PSC device. f) The photocurrent curves of the CH_3_NH_3_PbI_3_ solar cells with different P(VDF‐TrFE) doping concentrations. g–j) The statistic parameters of the solar cell performance.

We also noticed P(VDF‐TrFE) assembling behaviors when the concentration was further added to (>5 wt%). Large insulated P(VDF‐TrFE) particles were randomly spread on the surface, which led to a significant promotion of roughness, as shown in Figure S3 in the Supporting Information. This was in close connection to the upper layer (Spiro‐MeOTAD) (HTL) coverage decreasing, which was unfavorable for harvesting excellent devices with high fill factors (FFs). The performances of the FE‐PSCs were tested by current density versus voltage (*J–V*) scan measurements. Figure 4f shows four champion solar cells recorded in the reverse scan direction at 50 mV s^−1^ (1.2 V→‐0.2 V). There needed to be an optimized concentration of P(VDF‐TrFE) for the maximized PCE for the trade‐off among the enhanced build‐in electric field, the increased series resistance (*R*_s_), and the increased pinholes led by extra roughness. We continuously tuned the concentration of P(VDF‐TrFE) from 0 to 2 wt% to investigate the performances and found that the optimized doping concentration of P(VDF‐TrFE) was ≈0.5 wt%. Figure 4c shows the statistic results of the devices parameters, including the *J*_SC_ (short circuit current density), *V*_OC_, FF, and PCE with different P(VDF‐TrFE) concentration sin precursor solvents. When there were no P(VDF‐TrFE) materials doped in the CH_3_NH_3_PbI_3_ devices that only used the same relatively low concentration (0.65 × 10^−3^ m mL^−1^) of perovskite precursor exhibited the lowest *V*_OC_ (1.08 V), while using a precursor solvent with mixed 0.5 wt% P(VDF‐TrFE), leading to the highest *V*_OC_ up to over 1.17 V. The enhancement of the *V*_OC_ can be understood by considering the photovoltage loss reduction in the PSCs. Generally, the *V*_OC_ values were limited by the energetic alignment at the interfaces between the perovskite and the transport layers (HTL and ETL). This meant that the *V*_OC_ was partially decided by the lowest unoccupied molecular orbital (LUMO) level of the ETL and the highest occupied molecular orbital (HOMO) level. In our study, the ETL and the HTL were unchanged but the *V*_OC_ could be raised by as much as ≈110 mV via 0.5 wt% P(VDF‐TrFE) doping even before poling. This was due to the partial spontaneous polarization of the P(VDF‐TrFE) that often occurred during the crystalline process, which was confirmed by the PFM study shown in Figure 2c. However, a higher doping concentration of P(VDF‐TrFE) did not raise the *V*_OC_ further but rather decreased it. One reason for this was that the series resistances (*R*_s_) of P(VDF‐TrFE) of the doped devices became larger with an increasing doping concentration due to the insulating nature of the P(VDF‐TrFE) materials. The second reason was that introducing P(VDF‐TrFE) could also roughen the CH_3_NH_3_PbI_3_ film surface and increase the occurring rate of pinholes, causing the shunt resistances (*R*_sh_) to decrease. The final results of *R*_s_ increasing and *R*_sh_ decreasing caused reduced FF and *V*_OC_, which agreed with the trends of the series of *J–V* measurements. Additionally, we confirmed the passivation effect of the P(VDF‐TrFE) on the perovskite films with Electrochemical Impedance Spectra, as seen in Figure S4 in the Supporting Information. In a comprehensive way, the photovoltaic performances of the FE‐PSCs were the integrated outcome the ferroelectric field and the chemical passivation, extra *R*_s_ and reduced *R*_sh_ factors that were introduced by the P(VDF‐TrFE) dopants.

Figure 4e displays a cross‐section of a sample, illustrating a compact but relative thinner active perovskite layer (<160 nm) for a 0.65 m concentration of perovskite precursor solvent, which explains why the photocurrent was low, ≈20 mA cm^−2^. The absorber layer was forced to be thin because of the P(VDF‐TrFE) soluble issues and it was the biggest challenge to the enhancement of the device performance in order to exceed traditional PSC efficiency. However, in other respects, FE‐PSCs could be used in semi‐transparent solar cell applications and the high *V*_oc_ (1.17 V) for the MAPbI_3_ system is still significant.

We explored the influence of the poling process on the device performance for 2 wt% P(VDF‐TrFE) doped CH_3_NH_3_PbI_3_ films before the HTL was deposited, as shown in **Figure** **5**a. Voltages of 5 and 10 V were applied for 10 min on the ferroelectric films with both positive (under a reverse bias for the PSCs) and negative directions. After the HTL and Au electrodes were deposited, the *J–V* characteristics were measured (Figure 5b). The applied field (estimated ≈± 50 MV m^−1^) correlated well with the reported P(VDF‐TrFE) polarization coercive field value. In the poling process, applying a positive voltage (≈10 V) would add an extra electric field to the active P(VDF‐TrFE) doped perovskite layer, which had the same field direction as the electric field generated in the p–i–n junction. In consequence, the *V*_OC_ increased from ≈1.05 ± 0.02 to 1.15 ± 0.02 V after positive poling. While applying a relatively low negative voltage (5 V) to avoid burning, the *V*_OC_ decreased to about 1 V. The change of performance obtained by positive poling or negative poling can be understood by the reduced charge recombination in the devices with P(VDF‐TrFE) doped, which could introduce an extra electric field that would be expected to increase the drift length and the bimolecular recombination lifetime of the minority carriers. The deduction was also confirmed by photoluminescence (PL) lifetime measurements. We used probed exciton dynamics of the active‐layers as well as p–i–n layers with time‐resolved photoluminescence (TRPL) lifetime measurements to investigate how the P(VDF‐TrFE) affected the PL lifetime. As shown in Figure 5c, the PL quenching in the positive poled ITO|SnO_2_| CH_3_NH_3_PbI_3_|SpiroMeOTAD film was faster than that in the unpoled film, and much faster than that of negative poled film, confirming that positive poling helped the charge carriers to transfer from the active layers into the transport layers. From the deconvolution of the time‐resolved PL decays with a triexponential function, the average PL lifetimes (τ_av_) of the three devices are listed in Table S1 in the Supporting Information. Figure 4d implies that the existence of P(VDF‐TrFE) promoted the τ_av_ of the FBL after poling, which was in accordance with the fact that P(VDF‐TrFE) had a passivation effect as a dopant. From Figure 5b, the unique characteristic of ferroelectric coupling solar cells can be determined, which is that the photocurrent and open‐circuit voltage could be switched by the bias poling direction.

**Figure 5:**
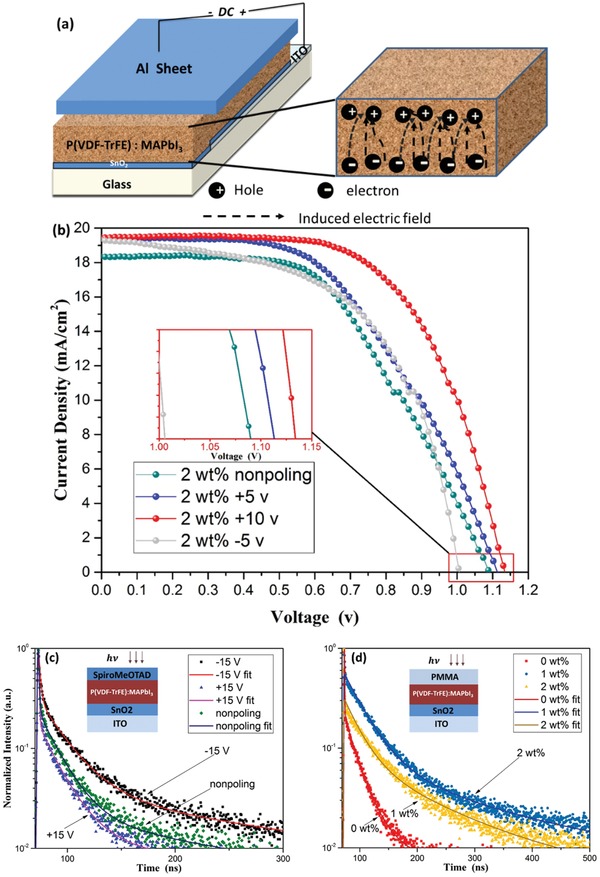
a) Illustration of the poling process and the electric‐field distribution and electron conduction through the ferroelectric P(VDF‐TrFE) doped CH_3_NH_3_PbI_3_ on the ETL side. b) Photocurrents of 2 wt% P(VDF‐TrFE) doped CH_3_NH_3_PbI_3_ solar cells before and after positive and negative poling. c) Time‐resolved photo‐luminescence results of the carrier extraction devices (p–i–n) before and after positive and negative poling. d) Time‐resolved photoluminescence results of CH_3_NH_3_PbI_3_ with various P(VDF‐TrFE) doping concentration films.

The ferroelectric coupling effect was first introduced into PSCs in this work, and the ferroelectric effect enhanced FE‐PSCs consisted of a ferroelectric material P(VDF‐TrFE) mixed into a CH_3_NH_3_PbI_3_ active layer using a low‐cost and feasible solution route. The ferroelectric coupling photovoltaic effect was revealed using both experimentation and a suitable simplified theoretical analysis with FE‐PSCs. This effect of ferroelectric material doping resulted in promoting the built‐in electric field, increasing both the diffusion and the drift‐driven charge transport and collection as well as reducing the nonradiative recombination loss, which was directly verified using PL lifetime measurements. Compared to typical CH_3_NH_3_PbI_3_‐based PSCs, these optimized FE‐PSCs presented high *V*_OC_s, up to 1.17 V, and PCEs up to 18%. The simplicity of adding P(VDF‐TrFE) dopants to a perovskite active layer and the excellent *V*_OC_ results also indicate that the FE‐PSC was a new successful application. However, we must note that the photocurrent of the PSCs doped with P(VDF‐TrFE) was relatively low because of the thinner absorber layer. Further work is necessary to reconcile both the thickness of the absorber layer and the doping ferroelectric materials, to take full advantage of the electric field for increasing charge‐collection efficiencies, and to ensure the proper absorption length for incident light.

## Conflict of Interest

The authors declare no conflict of interest.

## Supporting information

Supplementary
