# Supplementary material for: Efficiency Enhancement with the Ferroelectric Coupling Effect Using P(VDF‐TrFE) in CH3NH3PbI3 Solar Cells
Source: Adv Sci (Weinh). 2019 Jul 4;6(16):1900252. doi: 10.1002/advs.201900252 (PMC6702631; doi:10.1002/advs.201900252)
Supplement: Supplementary file 1 — Supplementary [file ADVS-6-1900252-s001.pdf]

Copyright WILEY-VCH Verlag GmbH & Co. KGaA, 69469 Weinheim, Germany, 2019.

Supporting Information

for *Adv. Sci.,* DOI: 10.1002/advs.201900252

Efficiency Enhancement with the Ferroelectric Coupling Effect Using P(VDF-TrFE) in $CH_3NH_3PbI_3$ Solar Cells

Endong Jia, Dong Wei, Peng Cui, Jun Ji, Hao Huang, Haoran Jiang, Shangyi Dou, Meicheng Li, Chunlan Zhou,* and Wenjing Wang*

Supporting Information

Efficiency Enhancement by Ferroelectric Coupling Effect Using P(VDF-TrFE) in Perovskite Solar Cells

Endong Jia, Dong Wei, Peng Cui, Jun Ji, Shangyi Dou, Meicheng Li, Chunlan Zhou*, Wenjing Wang*

E. Jia, Prof. C. Zhou, Prof. W. Wang
The Key Laboratory of Solar Thermal Energy and Photovoltaic System
Institute of Electrical Engineering
Chinese Academy of Sciences
Beijing, 100190, P. R. China
E. Jia, Prof. C. Zhou, Prof. W. Wang
University of Chinese Academy of Sciences (UCAS)
Beijing, 100049, P. R. China
E-mail: chunlanzhou@gmail.com
E. Jia, D. Wei, P. Cui, J. Ji, S. Dou, Prof. M. Li
State Key Laboratory of Alternate Electrical Power System with Renewable Energy Sources
School of Renewable Energy
North China Electric Power University
Beijing, 102206, P. R. China
E-mail: mcli@ncepu.edu.cn

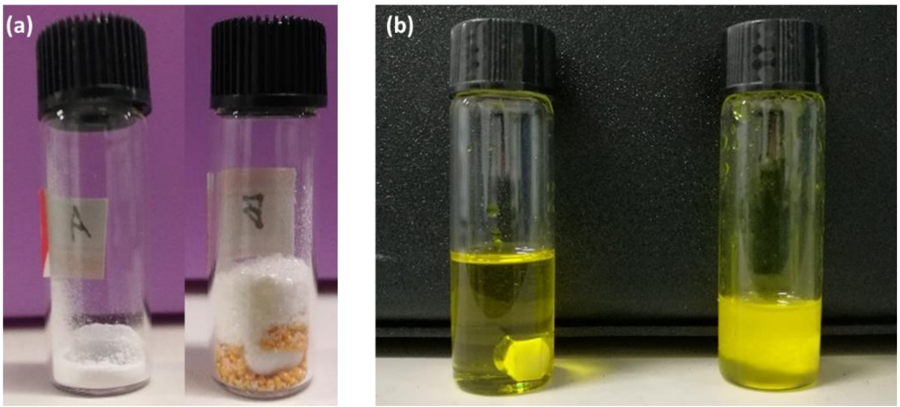

Figure S1. a) Pictures of white P(VDF-TrFE) powder, MAI mixed PbI_2 powder. b) 5 wt% P(VDF-TrFE) in 0.6 M PbI_2/DMF solution (left), 0.1 wt% P(VDF-TrFE) in 1 M PbI_2/DMF solution (right), after being stirred overnight.

Note:

Precursor solution with higher precursor concentraion (more than 0.65 M), any P(VDF-TrFE) could not be solved. Therefore, all perovskite samples used 0.5 M PbI2/MAI for precursor solution.

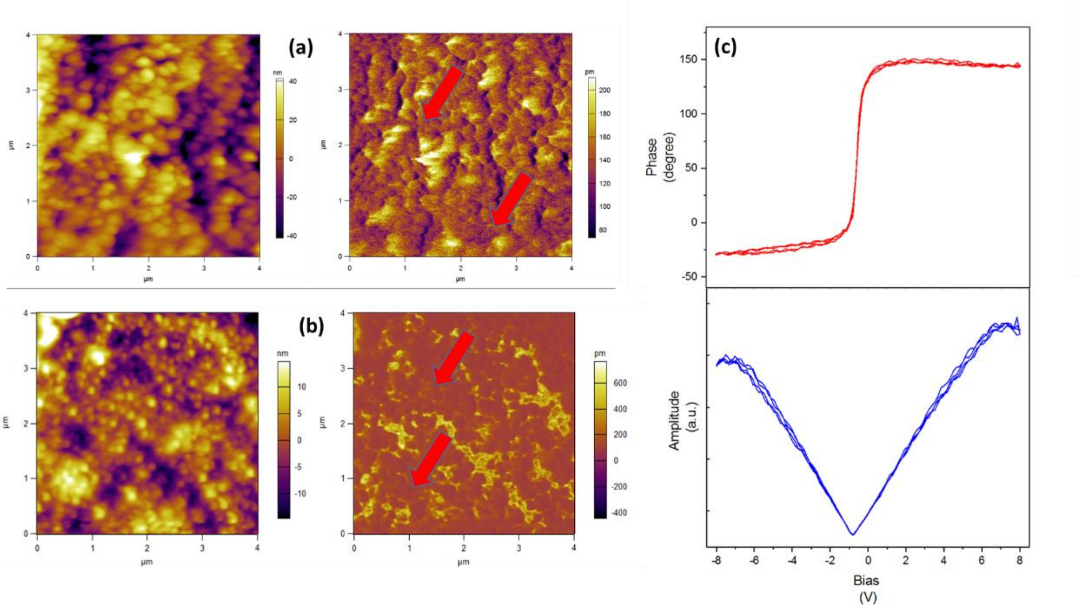

Figure S2. a) AFM topology and PFM amplitude images of pure $MAPbI_3$ film. b) AFM topology and PFM amplitude images of P(VDF-TrFE) doped $MAPbI_3$ film. c) No hysteresis loop (phase and amplitude) of the area of either the arrow of a) or b).

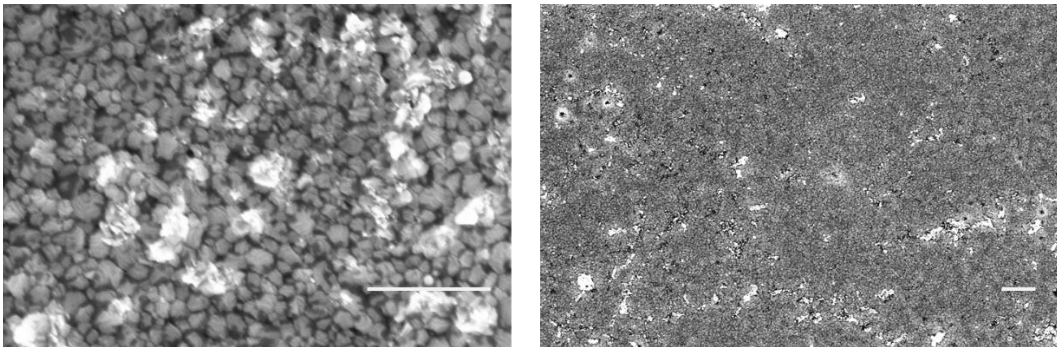

Figure S3. Top surface morphologies of the $CH_3NH_3PbI_3$ doped with 6 wt% P(VDF-TrFE) concerntration, the actual length of the white segements is 2 μm.

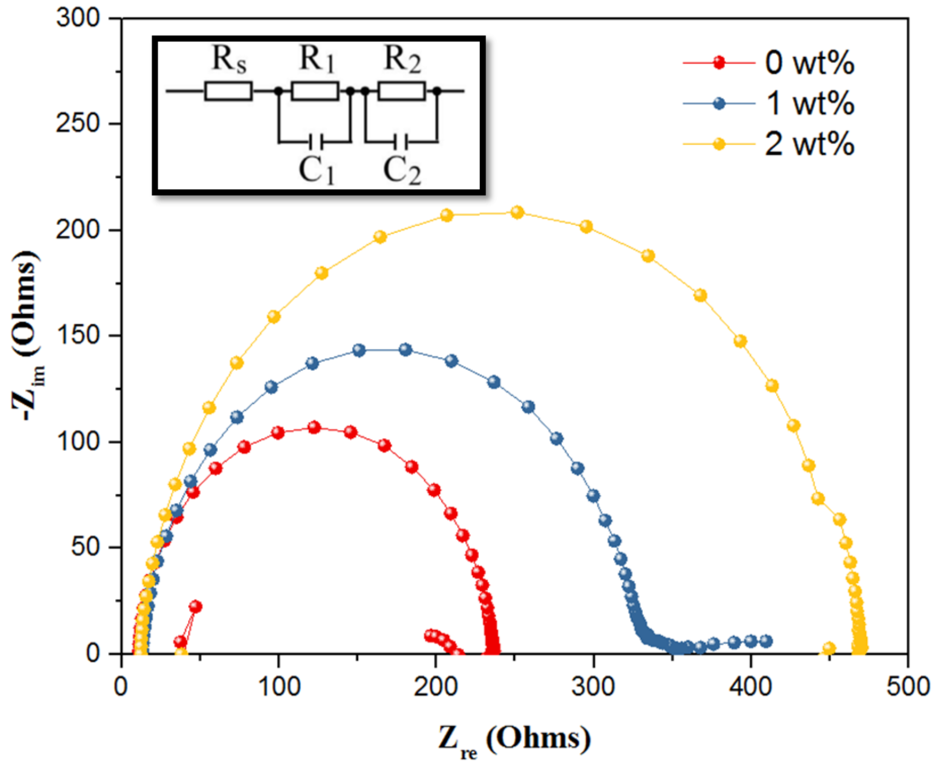

Figure S4. The EIS results indicate that the perovskite solar cells with larger concertration of P(VDF-TrFE) assisted perovskite film have a higher charge transfer rate and a lower carrier recombination rate which induces enhanced photovoltaic performance.

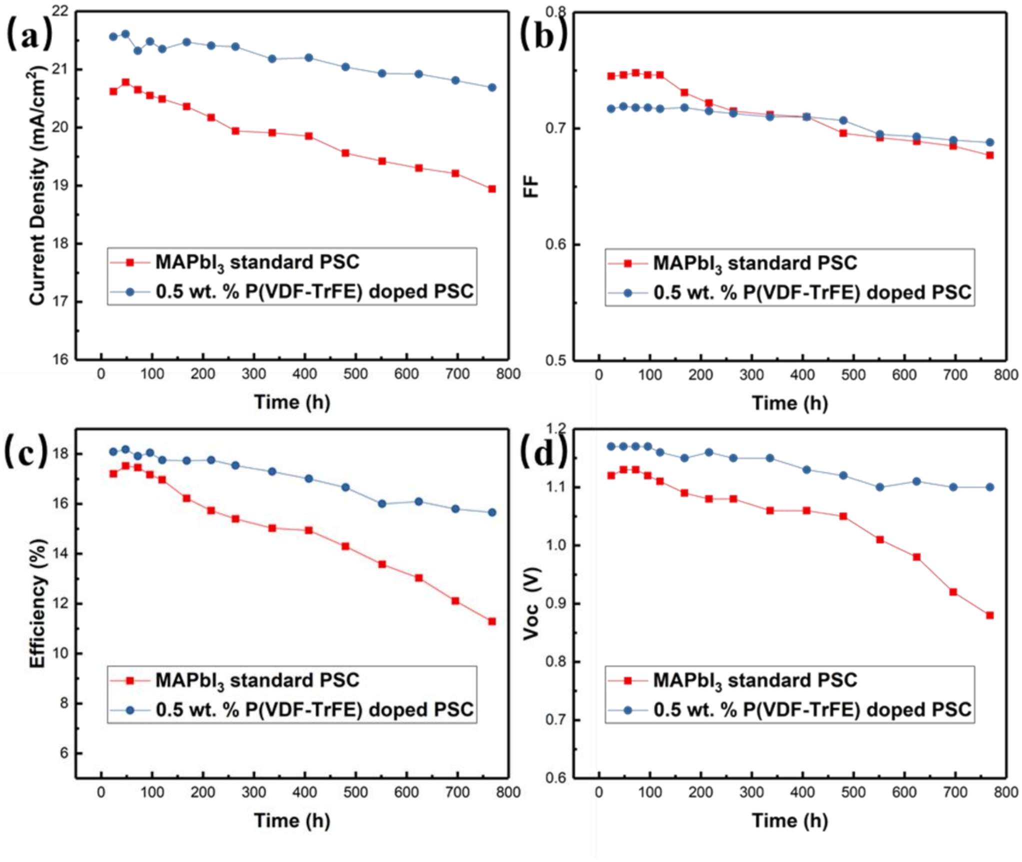

Figure S5. The stability trace of the standard MAPbI3 PSC and P(VDF-TrFE) doped MAPbI3 PSC. Evolution of J_{sc}**(a)**, fill factor**(b)**, conversion efficiency**(c)**, V_{oc}**(d)**. All devices were not encapsulated and stored in ambient atmosphere (25 °C, 45±10 %RH) under dark condition.

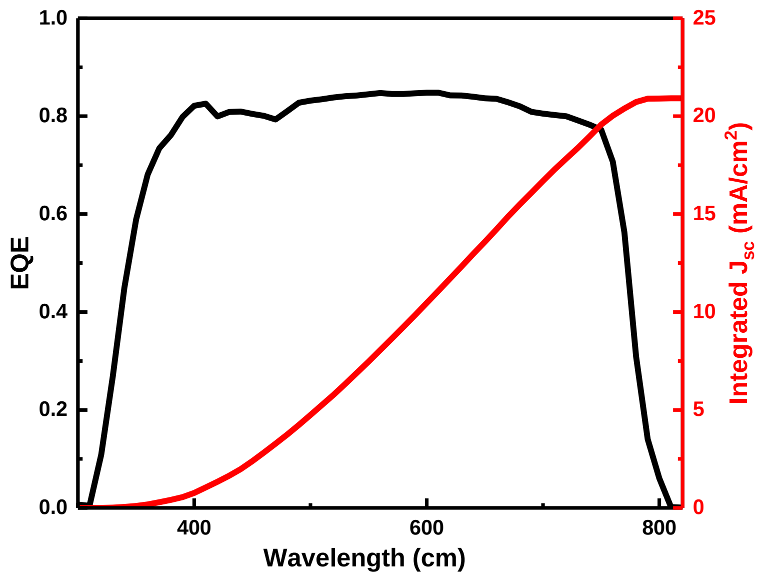

Figure S6. External quantum efficiency of the best device of 0.5 wt.% P(VDF-TrFE) doped MAPbI3 PSC. EQE spectrum is marked as black and EQE-based integrated short-circuit current density is marked as red, which is of the best efficiency 17.92%.

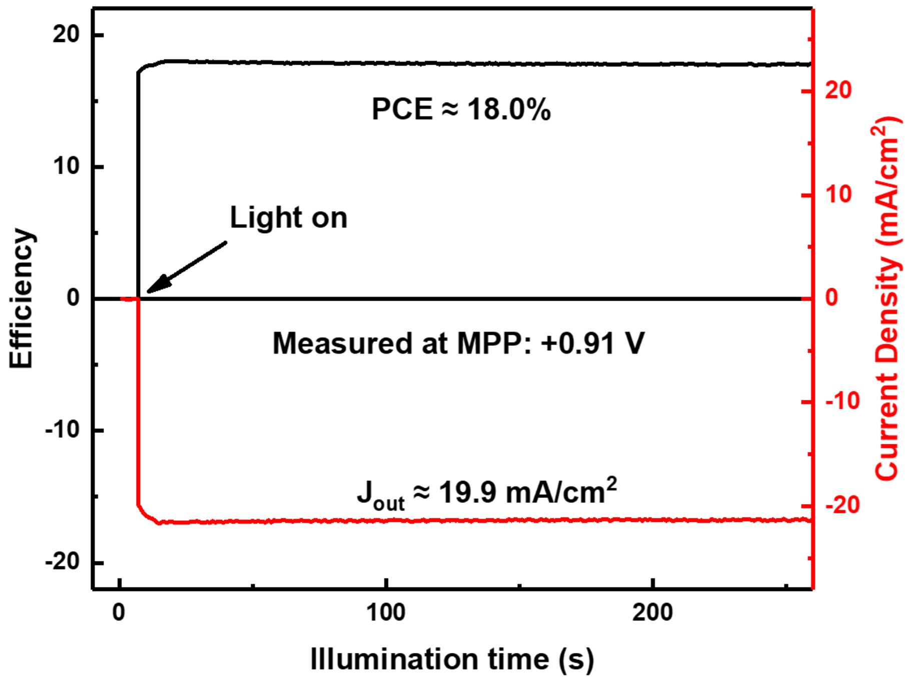

Figure S7. Steady-state photocurrent and efficiency at the maximum power point (MPP: 0.91 V) for the best device of 0.5 wt. % P(VDF-TrFE) doped MAPbI3.

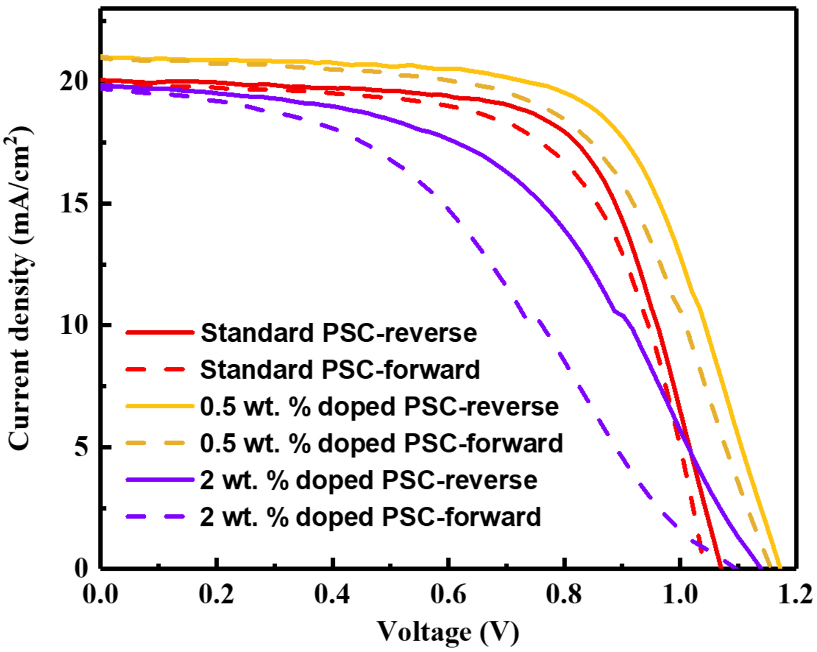

Figure S8. Hysteresis testing for the best devices of controlled undoped MAPbI3 PSC group and 0.5 wt. %, 2 wt. % P(VDF-TrFE) doped PSC group.

Table S1. Fitted parameters of TRPL curves for the samples doped with P(VDF-TrFE) treated by various poling process. (-15 V, +15V, nonpoling; sample structure is FTO/SnO_2/perovskite/spiro-MeTAD)

sample	A_1	τ_1(ns)	A_2	τ_2(ns)	τ_{avg}(ns)
-15 V	0.87	1.14	0.13	25.42	4.30
+15 V	0.78	1.94	0.22	34.04	9.00
nonpoling	0.82	1.31	0.18	25.68	5.70

Experimental Section

Synthesis P(VDF-TrFE) Doped Perovskite Film: First, three solutions (A, B and C) were prepared. Solution A was a mixture of anhydrous DMF and DMSO (DMF:DMSO = 9 : 1 in volume ratio). Solution B comprised P(VDF-TrFE) (50 : 50 mol%; PiezoTech) in solution A at a concerntration of 40 mg/mL. Solution C contained MAI and PbI_2 in solution A at a same concerntration of 1.3 mol/L. Next, four film solutions (F_1, F_2, F_3 and F_4) were made for depositing P(VDF-TrFE) doped perovskite films by blending solution A, solution B and solution C at volume ratios of 1 : 0 : 1, 3 : 1 : 4, 1 : 1 : 2, 0 : 1 : 1. This allowed to have final concentration of perovskite same (0.65 mol/L) in all 4 solutions and different (0 mg/mL, 5 mg/mL, 10 mg/mL and 20 mg/mL) concentration of P(VDF-TrFE) respectively, with each solution having same ratio of DMF and DMSO (90 : 10 vol%) to ensure appropriate comparison and avoid possible repeated weighing errors for powders. After magnetically stirred for several hours, solution F_1, F_2, F_3 and F_4 were filtered and then spin-cast onto substrates. The spin rate was 1500 rpm for 20 s and 1000 µL of aether was poured onto the spinning film 10 s prior the end of spin-casting process. The as-prepared films were then thermally annealed at 140 °C for 30 min. Pure P(VDF-TrFE) film samples were obtained by solution B spin-casting at 5000 rpm for 20 s and suffering aether poured onto it in the same way as well as the annealing process. All the film deposition and annealing processes were performed in an N_2-filled glove box.

Ferroelectric Perovskite Solar Cells Fabrication: Indium-doped tin oxide (ITO; Asashi Glass Co., Ltd.) coated glass slides etched by laser were cleaned by sonication in detergent, acetone, ethanol, deionized water. The ITO substrates were then blow-dried with nitrogen and underwent an UV ozone treatment for 30 min prior to depostion of compact SnO_2 layer. A thin (≈20 nm) SnO_2 layer was deposited on top of the ITO by spin-coating SnO_2 colloidal olution at 4000 rpm for 30 s. Then, it was heated at 140 °C for 30 min in air. The SnO_2 precursor was prepared by diluting SnO_2 colloidal dispersion (tin(IV) oxide, 15% in H_2O, Alfa Aesar) with H_2O at a volume ratio of 1 : 7. The Spiro-OMeTAD solution was coated at 4000 rpm for 30 s, where 1 ml Spiro-OMeTAD/chlorobenzene (90 mg/ml) solution was employed with the addition of 45 µl Li-TFSI/acetonitrile (170 mg/ml) and 10 µl tBP. Finally, a Au back electrode was deposited by thermal evaporation at a pressure of 2×10^{-5} Pa. The active area was 0.1 cm^2.

Materials and Device Characterizations: Photocurrent-voltage curves were Keithley 2400 power sourcemeter under AM 1.5G illumnation at an intensity of 100 mW cm-2 (Newport solar simulator) with reference to a reference cell PVM 13 certified by National Renewable Energy Laboratory. Both PSCs and FE-PSCs had no encapsulation and were tested IV/PFM in ambient air (25 °C, ~40±10 % humidity) and employed with an aperture area of 0.07 cm^2 mask. The EQE was measured using Enli Tech QE-R systems. The morphologies of the annealed perovskite films with and without P(VDF-TrFE) dopant were characterized by scanning electron microscopy (SEM) (FEI SIRION 200). The chemical compositions and structures of the films

were analyzed by X-ray diffraction (XRD) (Bruker D8 Advance X-ray diffractometer, Cu-Kα radiation λ= 0.15406 nm). Timeresolved PL spectra were measured by F900 spectrometer (Edinburgh, England). PFM mearurements were performed with a commercial AFM system (Asylum Research, MFP-3D Infinity) equipped with an external phase-locked loop (Zurich Instruments, HF2LI-PLL). The images were recorded in tapping mode, with a conductive PtSi-FM probe (NANOSENSORS, Pt-coated Si with a frequency of ~75KHz and a nominal tip radius of ~25nm). The PFM testing sample chamber was filled with dry air, testing procedure was carried out in dark. The DC poling process via AFM was used to switch the polarization inside the ferroelectric films, which started with smaller tip bias, such as -2 V→-1 V→ 0 V→+1 V→+2 V, and the sweep was from negative voltage to positive voltage. The piezoresponse hysteresis loops, were obtained from amplitude, A(E) and phase, φ(E), using the equation PR(E) = A(E)*cos(φ(E)) in simple harmonic oscillator (SHO) fitting method from the PFM software. To avoid uncertain electrostatic effects, the PFM sample was grounded through the FTO substrate the tip was grounded through the cantilever holder circuit during the previous conducting measurement.
